# Accuracy Enhancement of Inertial Sensors Utilizing High Resolution Spectral Analysis

**DOI:** 10.3390/s120911638

**Published:** 2012-08-27

**Authors:** Aboelmagd Noureldin, Justin Armstrong, Ahmed El-Shafie, Tashfeen Karamat, Don McGaughey, Michael Korenberg, Aini Hussain

**Affiliations:** 1 NavINST—Navigation and Instrumentation Research Group, Department of Electrical and Computer Engineering, Royal Military College of Canada, Kingston, ON K7K 7B4, Canada; E-Mails: noureldin-a@rmc.ca (A.N.); armstrong.jlk41@gmail.com (J.A.); tashfeen.karamat@rmc.ca (T.K.); mcgaughey-d@rmc.ca (D.M.); 2 Civil and Structural Engineering Department, University Kebangsaan Malaysia, Bangi 43600, Malaysia; 3 Department of Electrical and Computer Engineering, Queen's University, Kingston, ON K7L 3N6, Canada; E-Mail: korenber@queensu.ca; 4 Electric, Electronics Systems Engineering Department, University Kebangsaan Malaysia, Bangi 43600, Malaysia; E-Mail: aini@eng.ukm.my

**Keywords:** INS/GPS, spectral analysis, FOS, navigation system

## Abstract

In both military and civilian applications, the inertial navigation system (INS) and the global positioning system (GPS) are two complementary technologies that can be integrated to provide reliable positioning and navigation information for land vehicles. The accuracy enhancement of INS sensors and the integration of INS with GPS are the subjects of widespread research. Wavelet de-noising of INS sensors has had limited success in removing the long-term (low-frequency) inertial sensor errors. The primary objective of this research is to develop a novel inertial sensor accuracy enhancement technique that can remove both short-term and long-term error components from inertial sensor measurements prior to INS mechanization and INS/GPS integration. A high resolution spectral analysis technique called the fast orthogonal search (FOS) algorithm is used to accurately model the low frequency range of the spectrum, which includes the vehicle motion dynamics and inertial sensor errors. FOS models the spectral components with the most energy first and uses an adaptive threshold to stop adding frequency terms when fitting a term does not reduce the mean squared error more than fitting white noise. The proposed method was developed, tested and validated through road test experiments involving both low-end tactical grade and low cost MEMS-based inertial systems. The results demonstrate that in most cases the position accuracy during GPS outages using FOS de-noised data is superior to the position accuracy using wavelet de-noising.

## 1. Introduction

In numerous applications, inertial navigation system (INS) and global positioning system (GPS) are two complementary technologies that can be integrated to provide reliable positioning and navigation information for land vehicles. In the event of loss, denial of use, or degradation of the GPS signal (*i.e.*, due to signal jamming in military electronic warfare operations, or due to signal blockage while driving through urban canyons), INS can be an invaluable source of redundancy [1]. INS is inherently immune to the signal jamming, spoofing, and blockage vulnerabilities of GPS, but the accuracy of INS is significantly affected by the error characteristics of the inertial sensors it employs [2]. Thus, the accuracy enhancement of inertial sensors is a subject of widespread research [3].

The process of inertial navigation computes position, velocity and attitude of a moving platform, with respect to an inertial frame of reference, by measuring its rotational motion (using gyroscopes) and translational motion (using accelerometers) and mathematically integrating the measurements through a procedure known as INS mechanization [2]. The inertial sensors employed in an INS have significantly complex short-term (high-frequency) and long-term (low frequency) noise characteristics that are produced by many different error sources.

During the INS mechanization process, these errors are compounded, resulting in increasingly inaccurate position and attitude over time. Despite having an INS/GPS integration algorithm (like Kalman filtering) to correct for INS errors, it is advantageous to enhance the INS solution prior to the data fusion process [3]. This requires pre-filtering (or de-noising) each of the inertial sensor signals before they are used to compute position, velocity and attitude. Presently, optimal low-pass filtering and wavelet de-noising techniques are used to eliminate or minimize short-term errors from the inertial sensor signals, but these techniques have had limited success in removing the long-term errors that are mixed with the true motion dynamics of the moving platform [4]. Both bias and scale factor instabilities are stochastic in nature and exist in the low frequency part of the inertial sensor signal [3,4]. Thus they seriously impact the overall system performance since they may be mixed with motion dynamics. In low cost systems (e.g., low end tactical grade and MEMS-based inertial systems), besides the long design time, it may not be possible to come up with accurate stochastic models to be employed inside Kalman filtering in order to effectively compensate for the effect of such long-term sensor errors.

The research reported herein aims at: (1) developing a novel inertial sensor accuracy enhancement technique that can remove some or all of the error components from inertial sensor measurements prior to INS mechanization and INS/GPS integration; (2) examining the effectiveness of the proposed method on real INS/GPS road test data; (3) comparing the results to other wavelet based pre-filtering techniques.

Wavelet de-noising is the current state of the art technique used in the accuracy enhancement of inertial sensors [3,4] and is therefore used as a standard of comparison in this research. The surveyed literature indicated that the Daubechies family of wavelets and soft thresholding employing the principles of Stein's Unbiased Risk Estimate (SURE) are typically used in pre-filtering inertial sensors.

The proposed technique employs the fast orthogonal search (FOS) algorithm [5,6] to estimate the low frequency spectrum, which contains the vehicle motion dynamics and long term noise, with a high frequency resolution and small number of frequency terms. The high-resolution spectral analysis capabilities of FOS will result in modeling the low frequencies with a small number of terms. The dynamic noise threshold of FOS should accept the vehicle motion dynamics while rejecting the long term noise since the motion dynamics will likely have higher power than the long term noise terms.

## 2. Fast Orthogonal Search (FOS)

The fast orthogonal search (FOS) algorithm [5–9] is a general purpose modeling technique, which can be applied to spectral estimation and time-frequency analysis. The algorithm uses an arbitrary set of non-orthogonal candidate functions *p_m_*(*n*) and finds a functional expansion of an input *y*(*n*) in order to minimize the mean squared error (MSE) between the input and the functional expansion.

The functional expansion of the input *y*(*n*) in terms of the arbitrary candidate functions *p_m_*(*n*) is given by:

(1) $$y(n)=\sum_{m=0}^{M}a_{m}p_{m}(n)+\varepsilon (n)$$

where *a_m_* are the weights of the functional expansion, and *ε*(*n*) is the modeling error.

By choosing non-orthogonal candidate functions, there is no unique solution for Equation (1). However, FOS may model the input with fewer model terms than an orthogonal functional expansion [5]. For example, the fast Fourier transform (FFT) uses a basis set of complex sinusoidal functions that have an integral number of periods in the record length [10]. For the FFT to model a frequency that does not have an integral number of periods in the record length, energy is spread into all the other frequencies, which is a phenomena known as spectral leakage [5,9,11]. By using candidate functions that are non-orthogonal, FOS may be able to model this frequency between two FFT bins with a single term resulting in many fewer weighting terms in the model [5,9].

FOS begins by creating a functional expansion using orthogonal basis functions such that:

(2) $$y(n)=\sum_{m=0}^{M}g_{m}w_{m}(n)+e(n)$$

where *w_m_*(*n*) is a set of orthogonal function derived from the candidate functions *p_m_*(*n*), *g_m_* is the weight, and *e*(*n*) is an error term.

The orthogonal functions *w_m_*(*n*) are derived from the candidate functions *p_m_*(*n*) using the Gram Schmidt (GS) orthogonalization algorithm. The GS algorithm starts by setting the first orthogonal function *w*_0_(*n*) equal to the first candidate function *p*_0_(*n*):

(3) $$w_{0}(n)=p_{0}(n)$$

The next orthogonal function *w*_1_(*n*) is found by subtracting a weighted value of *w*_0_(*n*) from the second candidate function *p*_1_(*n*) as given by:

(4) $$w_{1}(n)=p_{1}(n)-\alpha_{10}w_{0}(n)$$

where *α*_10_ is the GS weight. Now *w*_0_(*n*) and *w*_1_(*n*) are orthogonal to each other, so the correlation of these function should equal zero as given by:

(5) $$\bar{w_{1}(n)w_{0}(n)}=\bar{p_{1}(n)w_{0}(n)}-\alpha_{10}\bar{w_{0}^{2}(n)}=0$$

where the overbar indicates the average over the N samples of the function, $\bar{x}=\frac{1}{N}\overset{N-1}{\underset{n=0}{\text{∑}}}x(n)$

Solving Equation (5) results in:

(6) $$\alpha_{10}=\frac{\bar{p_{1}(n)w_{0}(n)}}{\bar{w_{0}^{2}(n)}}=\frac{\bar{p_{1}(n)p_{0}(n)}}{\bar{w_{0}^{2}(n)}}$$

Subsequent orthogonal functions are found by subtracting weighted values of all the previously fitted orthogonal functions from the kth candidate function as given by:

(7) $$w_{m}(n)=p_{m}(n)-\sum_{r=0}^{m-1}\alpha_{mr}w_{r}(n)$$

where the GS weights can be shown to be given by:

(8) $$\alpha_{mr}=\frac{\bar{p_{m}(n)w_{r}(n)}}{\bar{w_{r}^{2}(n)}}$$

The orthogonal functions *w_m_*(*n*) are implicitly defined by the Gram-Schmidt coefficients *α_mr_* and do not need to be computed point-by-point. Using the same procedure as in Equation (5), the Gram-Schmidt coefficients *α_mr_* can be found recursively using the equations [5,6]:

(9) $$D(m,0)=\bar{p_{m}(n)p_{0}(n)}$$

(10) $$D(m,r)=\bar{p_{m}(n)p_{r}(n)}-\sum_{i=0}^{r-1}\alpha_{ri}D(m,i)$$

and

(11) $$\alpha_{mr}=\frac{\bar{p_{m}(n)w_{r}(n)}}{\bar{w_{r}^{2}(n)}}=\frac{D(m,r)}{D(r,r)}$$

Note, it can be shown that [5,6]:

(12) $$\bar{w_{m}^{2}(n)}=D(m,m)$$

and this was used in simplifying Equation (11).

The next step in FOS is to compute the weights of the orthogonal functional expansion *g_m_* in Equation (2) that minimize the mean squared error between the functional expansion and the input *y*(*n*). The mean squared error is given by:

(13) $$\bar{\varepsilon^{2}(n)}=\bar{{\left(y(n)-\sum_{m=0}^{M}g_{m}w_{m}(n)\right)}^{2}}$$

By taking the derivative with respect to *g_m_* and solving, it can be shown that the values of the *g_m_* that minimize the MSE are given by:

(14) $$g_{m}=\frac{\bar{y(n)w_{m}(n)}}{\bar{w_{m}^{2}(n)}}$$

The correlation between the input *y*(*n*) and the orthogonal functions *w_m_*(*n*) can be found recursively using the equations:

(15) $$C(0)=\bar{y(n)p_{0}(n)}$$

and:

(16) $$C(m)=\bar{y(n)p_{m}(n)}-\sum_{r=0}^{m-1}\alpha_{mr}C(r)$$

Using Equation (12) and Equation (14), the weights *g_m_* that minimize the MSE of the orthogonal functional expansion can be found using:

(17) $$g_{m}=\frac{C(m)}{D(m,m)}$$

In its last stage, FOS calculates the weights of the original functional expansion *a_m_* (Equation (1)), from the weights of the orthogonal series expansion, *g_m_*, and the weights *α_ir_*. The value of *a_m_* can be found recursively using:

(18) $$\begin{array}{cc} a_{m}=\sum_{i=m}^{M}g_{i}v_{i}, & v_{m}=1 \end{array}$$

where

(19) $$\begin{array}{cc} v_{i}=-\sum_{r=m}^{i-1}\alpha_{ir}v_{r}, & i=m+1,m+2,\ldots ,M \end{array}$$

From Equations (9), (10), (15) and (16) it can be noted that FOS requires the calculation of the correlation between the candidate functions, and the correlation between the input and the candidate functions. The correlation between the input and the candidate function $\bar{y(n)p_{m}(n)}$ are typically calculated point-by-point once at the start of the algorithm and then stored for later quick retrieval. For regularly sampled data, the correlation between the candidate functions can be computed with closed form expressions, significantly reducing the number of computations required to compute these correlations [10,13].

Using the fact that the *w_m_*(*n*) are an orthogonal set of functions, the MSE of the orthogonal function expansion (Equation (13)) can be reduced to [5,6]:

(20) $$\bar{\varepsilon^{2}(n)}=\bar{y^{2}(n)}-\sum_{m=0}^{M}g_{m}^{2}\bar{w_{m}^{2}(n)}$$

It then follows that the MSE reduction given by the m^th^ model addition is:

(21) $$Q_{m}=g_{m}^{2}\bar{w_{m}^{2}(n)}=g_{m}^{2}D(m,m)$$

FOS can fit a model with a small number of model terms by fitting terms, which reduce the mean squared error (MSE) in order of their significance. The FOS search algorithm is stopped in one of three cases. The first is when certain maximum number of terms is fitted. The second case is when the ratio of MSE to the mean squared value of the input signal is below a pre-defined threshold. The third case is when adding another term to the model reduces the MSE no more than if it were fitting white Gaussian noise. In the case where FOS first fits a constant term (*p*_0_(*n*) = 1), the threshold test for determining if a new term (term m + 1) is not reducing the MSE by more than if it were fitting white Gaussian noise (WGN) is [6]:

(22) $$Q_{m+1}<\frac{4}{N}\left(\bar{y^{2}(n)}-\sum_{i=0}^{m}g_{i}^{2}\bar{w_{l}^{2}(n)}\right)$$

where *Q_m_*_+1_ is the MSE reduction of the new term and N is the number of points in the input data. A threshold test that does not assume a zero frequency term is fitted first can be found in [8].

Spectral analysis with FOS is accomplished by selecting candidates *p_m_*(*n*) that are pairs of sine and cosine terms at each of the frequencies of interest. The candidate functions *p_m_*(*n*) are given by:

(23) $$\begin{array}{c} p_{2m}(n)=\cos(\omega_{m}n)\\ p_{2m+1}(n)=\sin(\omega_{m}n) \end{array}$$

where *m* = 1, …, *P*, *ω_m_* is the digital frequency of the candidate pair and *P* is the number of candidate frequencies. By fitting a sine and cosine pair at each candidate frequency, the magnitude and phase at the candidate frequency can be determined [7,8].

There are at least two significant differences between FOS and the discrete Fourier transform (DFT) [5,6,10]: (1) FOS yields a parsimonious sinusoidal series representation by selecting the most significant sinusoidal components first; and (2) the frequencies of the sinusoids selected need not be commensurate nor integral multiples of the fundamental frequency corresponding to the record length. This translates to better frequency resolution in the spectral model.

FOS is appreciably better at rejecting coloured and white noise than the commonly used FFT techniques (example in [9]), which is significant since these types of errors are typically present in inertial sensor data.

## 3. Application of FOS to Inertial Sensor Accuracy Enhancement

In this research, FOS is used for inertial sensor accuracy enhancement. In general, signal de-noising typically involves: (1) transforming the data into a different domain (*i.e.*, wavelet transform or DFT) so that it can be represented by a series of weighted terms; (2) applying a thresholding method against the weighted terms to select only the most significant components, thus permitting the rejection of noise; and (3) performing an inverse transform on the selected significant terms to synthesize a time-series representation of the data with reduced noise. Although FOS is computationally demanding, the authors' research team was able to optimize it for real time realization [14–17]. The idea of utilizing FOS to de-noise inertial sensors was explored originally by the authors [14] but at much limited scope and experimentation.

FOS has been shown to be good at detecting the frequencies of interest buried in coloured and white noise even at signal to noise ratios (SNR) as low as −10 dB [9]. Coloured or correlated noise is consistent with the long term error characteristics present in inertial sensor outputs. The computation time required for FOS is considerably higher than that of the FFT or wavelet de-noising techniques, but with current-day personal computers the signals can still be processed in real-time.

### 3.1. Design of a FOS-Based Inertial Sensor Accuracy Enhancer

The basic principle behind the proposed FOS-based inertial sensor accuracy enhancer is to use FOS to model the motion dynamics measured by the inertial sensors, and reject as much of the inertial sensor error components as possible. As FOS models the noisy input data, it implicitly performs the aforementioned tasks of transforming and thresholding. FOS performs thresholding using Equation (22), when it evaluates whether the addition of a candidate function will reduce the MSE more than if it were fitting white noise. The inverse transform is a simple matter of using the model terms selected by FOS to synthesize a time-series using Equation (1).

**Figure 1.**
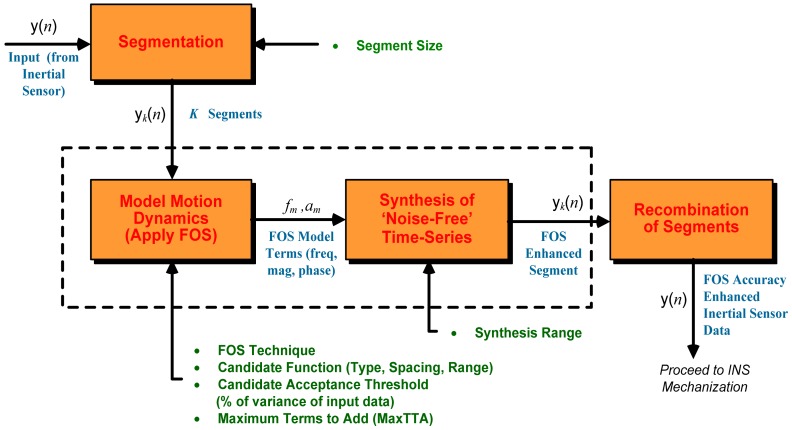
Block diagram of FOS-based inertial sensor accuracy enhancement technique (bullets denote operating parameters).

It should be noted that this process of de-noising is appropriate for stationary data (the frequency content of the data does not change with time), and that inertial sensor data is typically non-stationary since they are measurements of the vehicle motion dynamics and the sensor noise is also known to be non-stationary. The challenge of de-noising non-stationary inertial sensor data is addressed by taking segments of inertial sensor output and applying the FOS to each segment. Figure 1 shows a schematic of the FOS technique developed in this research.

The FOS accuracy enhancement technique initially segments a noisy input time series, denoted *y*(*n*) representing one of the six inertial sensor outputs, into smaller analysis windows that can be treated as stationary data. Each segment is modelled using FOS to extract the components of the motion dynamics from the noisy measurements. The output of this stage is the FOS model terms, which provides information on frequency, magnitude and phase for that segment of data. The FOS model terms can then be used to synthesize an estimation of the true motion dynamics for that segment, thus creating an accuracy enhanced segment. This process repeats for all segments, and all of the enhanced segments are recombined to create the overall accuracy enhanced inertial sensor time series.

### 3.2. FOS Accuracy Enhancement Parameters

As FOS is generally known to be a data dependent algorithm [5–10], the accuracy of the model produced by FOS depends on the data record being modelled, the candidate functions used to compute correlations, and the stopping conditions (thresholds) in the algorithm.

Sinusoidal candidate functions were selected in this research because they had been successfully applied to de-noising [5,6] and in non-stationary signal analysis [18]. Furthermore, the closed-form expressions for computing the cross-correlations between sinusoids [13] make the execution time of FOS with sinusoidal basis functions considerably faster than it would be with most other types of basis functions which require the correlations to be computed point by point [5].

The FOS candidate frequencies are chosen to have a higher resolution than the fast Fourier transform (FFT) to achieve better de-noising. The frequency resolution of an FFT can be given by:

(24) $$\text{FFT}\;\text{Resolution}=f_{S}/N$$

where *f_S_* is the sampling frequency and *N* is the number of points in the record. Subject to the SNR, it has been shown that FOS can achieve frequency resolutions up to 5 [10], 8 [6,12,18], or 10 [8] times the frequency resolution of the FFT.

From Equation (24) it can be seen that a long record length gives good spectral resolution, which is needed to accurately model a time-series. However, as inertial sensor data is time-varying, a short record length is desired for good time resolution on the time varying parameters. For this research, the candidate function spacing was typically set in the order of 1/8 the FFT resolution for each segment.

Candidate frequencies can be selected so that the candidate functions focus on a particular frequency range of interest. For example, the candidates can be spaced with a high resolution on a range of interest and outside the range of interest, the candidates can be spaced by FFT resolution intervals.

It is desirable to have the minimum number of candidate frequencies in a model required to model the motion dynamics. Too few terms results in a model that does not accurately model the input signal. Too many terms will add noise terms into the motion dynamics model as well as increase the computation time. In this research, the maximum number of frequencies to add (MAXFTA) is typically set between 6 and 15, not including the initial zero frequency model term.

FOS stops modelling when adding a new frequency pair does not reduce the MSE more than fitting WGN. It is known that INS data includes WGN and coloured noise, which may not be rejected by this threshold. Thus, a candidate acceptance threshold, requiring a frequency pair to fit a minimum percentage of the overall energy in the signal, is set [5]. Although there is no way of telling whether a FOS term models noise or motion dynamics, FOS will fit a small number of frequency terms rejecting WGN and terms with a small percentage of the overall energy, hopefully selecting the motion dynamic terms and rejecting the long term noise terms.

Finally, in synthesizing the noise free motion dynamics, FOS can synthesize the time-series estimates using only a certain range of frequencies detected by FOS. Since it is known that motion dynamics are typical low frequencies, (e.g., between 0 and 3 Hz for land vehicles in benign environments), FOS can model the motion dynamics with only model terms in this frequency range while rejecting the higher frequency terms as noise. This has the effect of low-pass filtering, thereby eliminating the short term error components of the inertial sensor measurements. Using FOS differs from using a low-pass filter (LPF) in that it has an adaptive noise rejection threshold and only fits a small number of frequency terms, whereas a LPF will pass all frequencies within its passband.

## 4. Simulation

A set of simulated inertial signals (gyroscopes and accelerometers in x, y, z axes) was generated at a 75 Hz data rate to correspond to a 120 s land vehicle trajectory. The true motion dynamics were generated using windowed sinusoidal functions, and the data was processed using an INS mechanization algorithm to produce an error-free reference trajectory [19,20]. Short- and long-term errors were then added to the reference signals to create a ‘noisy’ set of data, which would then be processed using wavelet de-noising and FOS de-noising. From here on, the abbreviations REF, NSY, WDN and FOS may be used when referring to the reference, noisy (or corrupted by short- and long-term errors), wavelet de-noised and FOS de-noised signals or trajectories. The NSY inertial sensor signals were processed with the INS mechanization algorithm to produce the NSY trajectory.

The short-term error component is made up of white Gaussian noise with variance levels matching those observed from a low-end tactical grade IMU (TG-6000, KVH Industries Inc., Middletown, RI, USA). The long-term error component is created with a 1st order Gauss-Markov process with a standard deviation and correlation time similar to those observed from the TG-6000 IMU. The Gauss-Markov disturbance is representative of long term inertial sensor errors like the bias drift, and has a correlation time of 1 h.

As mentioned, the results of the FOS de-noising are compared to results of the wavelet de-noising. The parameters of the wavelet de-noising function include the type of wavelet basis function, the number of levels of decomposition (LOD), and thresholding rules. The Daubechies family of wavelets with soft thresholding based on Stein's Unbiased Risk Estimate (SURE) are used in this paper as these parameters are typically used in pre-filtering inertial sensors [3,4]. The number of levels of decomposition (LOD) for the Wavelet transform is selected based on the data rate of the inertial sensor outputs and the expected frequency range of the vehicle motion dynamics. Each level of decomposition limits the frequency bandwidth of the WDN output by a factor of 2. In the case of this simulation, the inertial sensor data sampled at 75 Hz has an effective bandwidth of 37.5 Hz, and *n* LOD will limit the frequency band of the WDN output to (37.5/2*^n^*) Hz. To ensure a fair comparison between the WDN and FOS accuracy enhanced results, the wavelet de-noising technique is applied separately to each segment of NSY data that are processed by FOS de-noising.

Both the FOS and Wavelet de-noising used 15 s segments. The wavelet de-noising parameters used are shown in Table 1. The band where most of the spectral energy of the true motion dynamics for the x-acc, y-acc and z-gyro is from 0 to approximately 0.6 Hz. Thus six LOD were chosen to limit the WDN output band to approximately 0.6 Hz (37.5/2^6^ = 0.586 Hz). Since it is known in this simulation that there are no motion dynamics present for the x-gyro, y-gyro and z-acc, these signals should be de-noised as much as possible by using the maximum LOD (10 in this case).

The FOS de-noising parameters used for this data are summarized in Table 2. Note that an iterative FOS algorithm [8] was used to improve the FOS models. The FOS candidates had eight times the resolution of the FFT in the frequency band where motion dynamics were expected and used the FFT frequency resolution spacing outside this band. For the x-gyro, y-gyro and z-acc where no motion is expected, the high resolution band for FOS candidate frequencies is from 0 to 0.0366 Hz.

**Figure 2.**
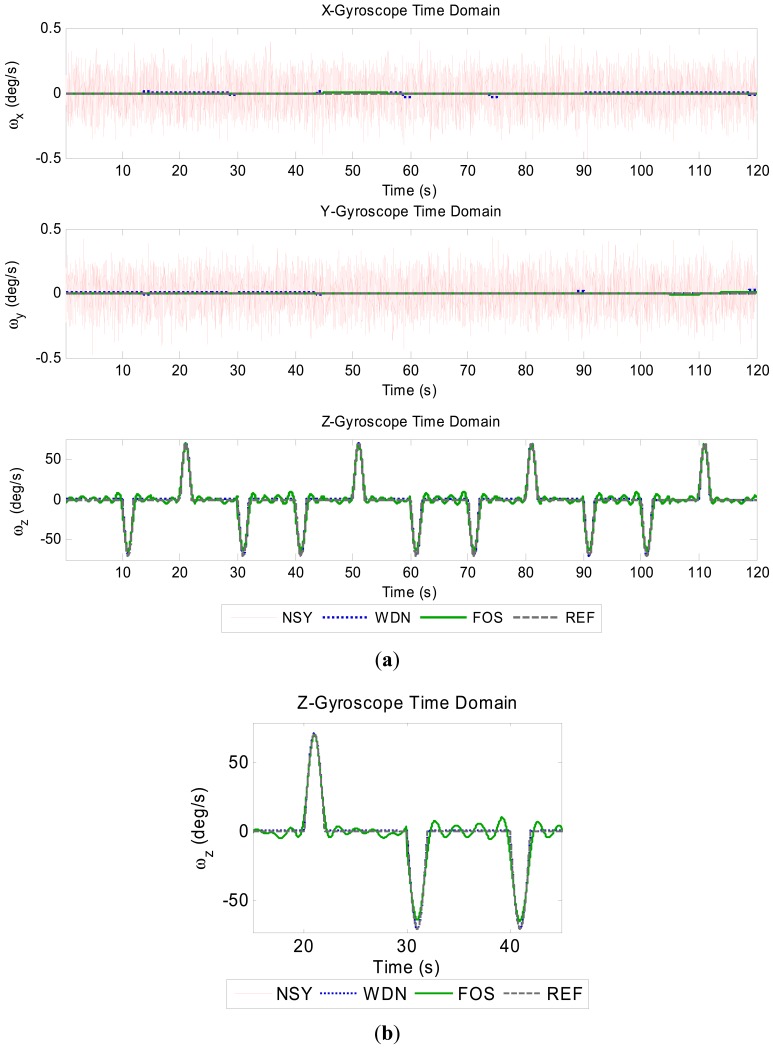
Accuracy enhanced gyroscope signals. (**a**) Gyroscope signals. (**b**) Zoomed-in section of z-gyro illustrating transients.

**Figure 3.**
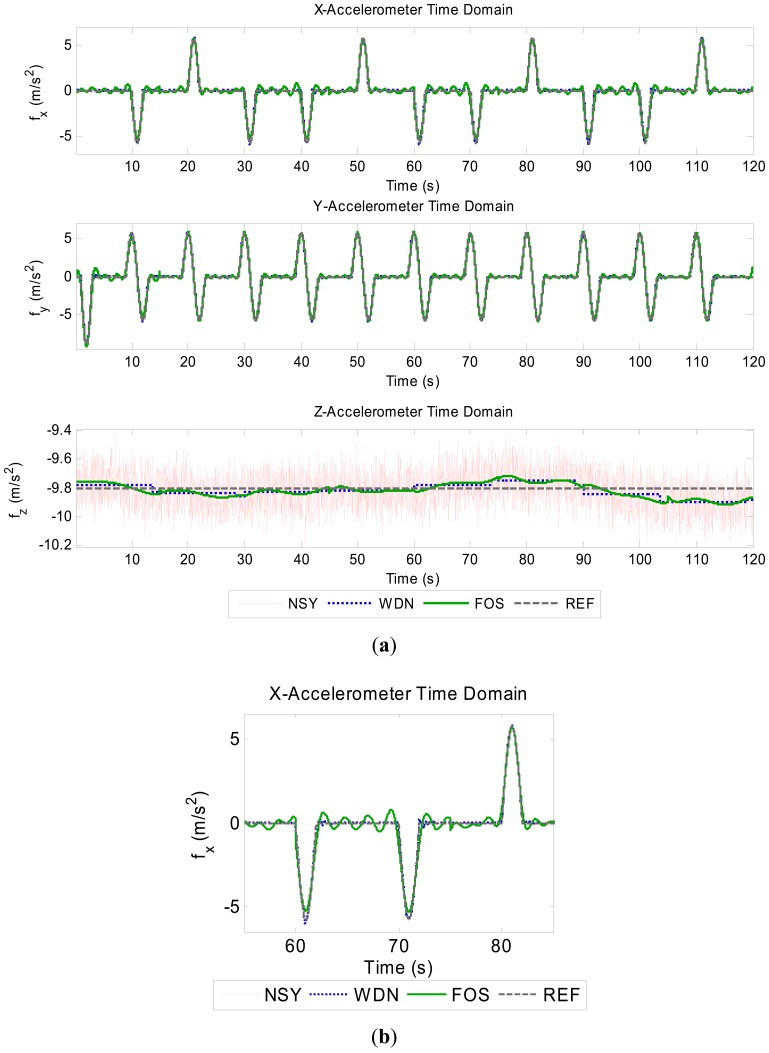
Accuracy enhanced accelerometer signals. (**a**) Accelerometer signals. (**b**) Zoomed-in section of x-acc illustrating transients.

**Figure 4.**
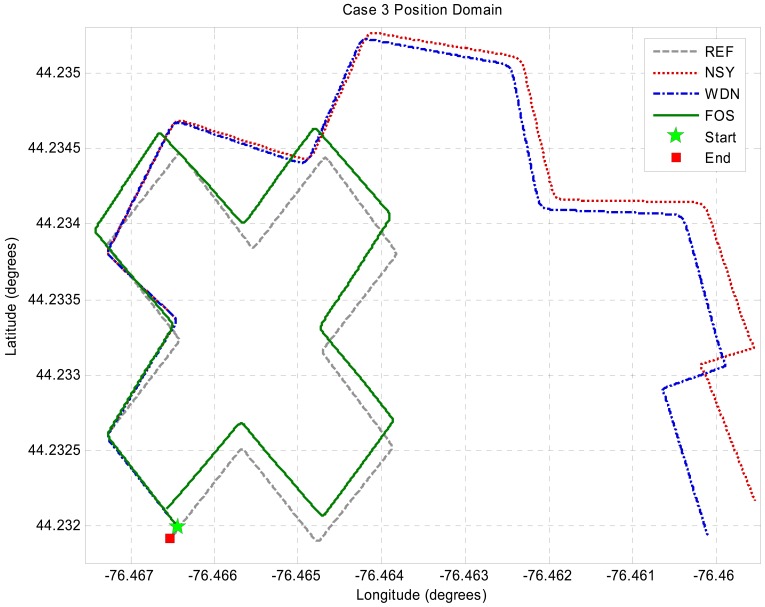
Trajectory plots.

The resultant accuracy enhanced signals for the gyroscopes and accelerometers are shown in the Figures 2 and 3. Since the FOS de-noised outputs (especially z-gyro, x-acc and y-acc around the zero-mean portions) use so few frequency terms, these signals can be seen to have ripple (Giibs phenomena) in them. However, the FOS de-noising position domain solution is vastly superior to the WDN solution, as shown in Figure 4. The INS mechanization process, through the mathematical integrations, integrates out the errors introduced by the ripple effect. It appears that the FOS de-noising is only fitting frequency terms associated with the vehicle motion dynamics and that long term noise components are not being modeled. The separation of the true motion dynamics from the long-term inertial sensor error components is a very difficult task. The motion dynamics are mixed in with the errors, and in practice it is impossible to determine whether or not FOS is inadvertently modeling the correlated error terms. In the simulation presented the position estimates using FOS de-noising were substantially better than with Wavelet de-noising suggesting that in this case FOS was only modeling motion dynamics. The potential of FOS is quite significant when one considers that the positions are computed without any external aiding (*i.e.*, no data fusion with GPS). The position errors from a low-end tactical grade INS may reach upwards of 30 m during a 30 s GPS outage. The resultant free-inertial trajectory from the simulated NSY data set reached a maximum horizontal position error of 204 m over 120 s, and accuracy enhancement from FOS reduces this error to 27.8 m (see Figure 4). Levels of accuracy enhancement like this for real inertial data would represent a significant breakthrough in inertial navigation and redundancy during loss of GPS. The maximum and mean horizontal position errors for the NSY, WDN and FOS trajectories for this simulation are summarized in Table 3.

### Experimental Work

**Figure 5.**
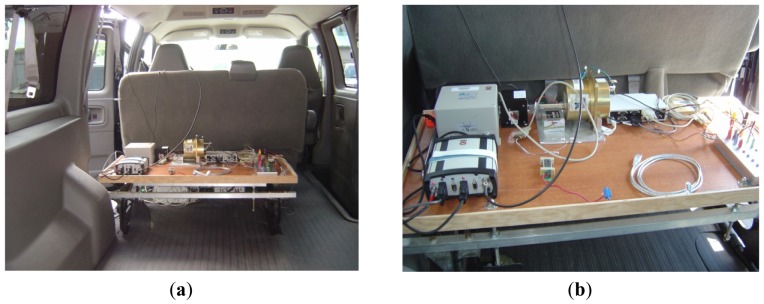
Experimental setup including inertial systems, GPS and data acquisition modules mounted inside land vehicle.

The impact of the proposed FOS method as a high-resolution spectral de-noising technique for inertial sensors is examined on two different types of inertial systems during two different road tests performed within the city of Kingston (Ontario, Canada) [21,22]. The experimental data collected in this research was completed using a van carrying a suite of measurement equipment that included inertial sensors, GPS receivers and antennas, computers to control the instruments and acquire the data as well as the required power supplies and connectors. A photograph of the experimental set-up is provided in Figure 5. The photos in Figure 5 show more equipment than those used in the experimental work of this paper.

The first experiment used a TG-6000 tactical grade IMU (KVH Industries, Inc.) and an 8-channel continuous-tracking GPS receiver with antenna (Lassen SQ GPS, Trimble Navigation Ltd., Sunnyvale, CA, USA). The inertial system used in the second road test is the Crossbow MEMS grade IMU (Crossbow Technologies, San Jose, CA, USA). Some specifications of both IMUs and GPS module are provided in Table 4.

In this study, for both the tactical grade and MEMS based inertial systems, the raw noisy inertial sensor measurements (denoted as NSY data) is processed by both the wavelet de-nosing procedure [4] and the FOS [5]. The inertial sensor measurements de-noised using wavelet analysis and FOS are denoted as WDN data and FOS data, respectively. The de-noising is followed by INS mechanization and a de-centralized fusion with GPS. The INS/GPS integration was accomplished through a KF-based data fusion software known as Aided Inertial Navigation System (AINS; Mobile Multi-Sensor Research Group, Department of Geomatics Engineering, University of Calgary, Alberta, Canada). The AINS software performs the following tasks: INS mechanization of the inertial sensor data; optimal estimation of navigation solution errors and sensor errors using a 15-state KF; and simulation of GPS outages. The data acquired from the Lassen SQ GPS module was applied to AINS as the GPS external aiding measurements while the NSY, WDN, and FOS data were applied as the IMU measurements.

One of the objectives of this experimental work is to assess the position domain accuracy enhancement of FOS during a GPS outage. Although there were no GPS outages during each of the above road tests, several 30 s GPS outages were intentionally introduced within each trajectory. The raw inertial sensor data and the GPS measurements were processed without applying any GPS outages to create a baseline AINS position domain solution and may be denoted REF from hereon. The AINS position solutions for the NSY, WDN, and FOS inertial sensor data sets during the outages are compared against the REF trajectory. The corresponding position errors during the outages can be used to assess FOS as an accuracy enhancement technique for a real INS/GPS integrated system.

## 5. Results and Discussion

### 5.1. Tactical Grade IMU

**Figure 6.**
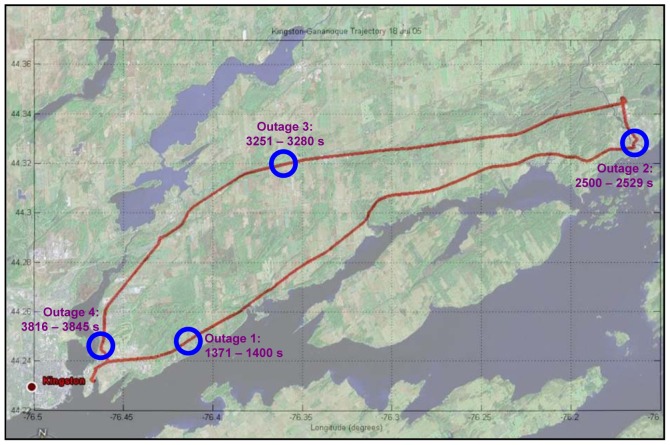
The first road test trajectory (for the TG6000 IMU) with the location of some intentionally introduced GPS outages indicated.

The trajectory of the first road test is shown in Figure 6. The TG6000 inertial sensors measurements were acquired at 75 Hz data rate. A segment size of 150 data points together with 75 Hz sampling rate corresponds to a 2 s record length.

**Figure 7.**
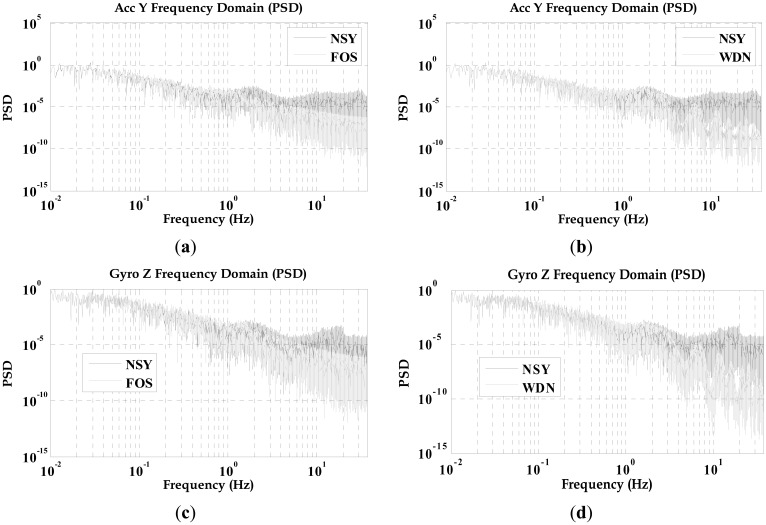
PSD plots for TG6000 inertial sensors before and after applying both FOS and WDN.

The WDN and FOS signals followed the trends of the NSY signals, but the WDN and FOS results had noticeably less short-term error components (*i.e.*, high-frequency components, white noise). Analysis in time-domain showed that the FOS data (although less noisy) follows the trends of the NSY and WDN data, thus demonstrating that FOS can operate on real inertial sensor data. Figure 7 compares the power spectral density (PSD) of the raw measurements before and after de-nosing utilizing both WDN and FOS. Figure 7(a,b) compare, respectively, the PSD of the NSY to FOS and the NSY to WDN for the entire trajectory for the forward axis accelerometer data while Figure 7(c,d) show the PSDs for the vertical gyroscope data. It can be depicted that after processing the raw measurements by FOS, they are noticeably less noisy than the NSY signals for both sensors. From Figure 7, it can be also determined that FOS can provide better attenuation of noise starting for the low frequency part of the signal (between 1 and 2 Hz) and also for the higher frequency band than WDN. WDN was not able to provide any noise suppression in the low frequency part where correlated long-term errors were mixed with part of the motion dynamics. Since examining the PSD plots alone cannot verify that FOS was more capable than WDN in removing long-term errors between 1 and 2 Hz, we decided to examine the impact of both FOS and WDN in the positioning accuracy.

**Figure 8.**
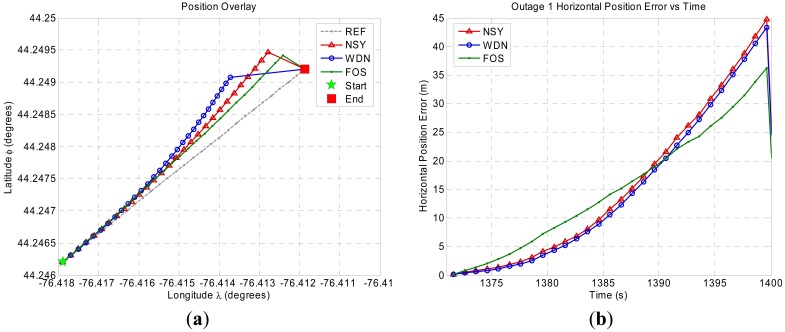
Land vehicle experiment position domain results for GPS outage 1. (**a**) Position domain plot. (**b**) Horizontal position error plot.

**Figure 9.**
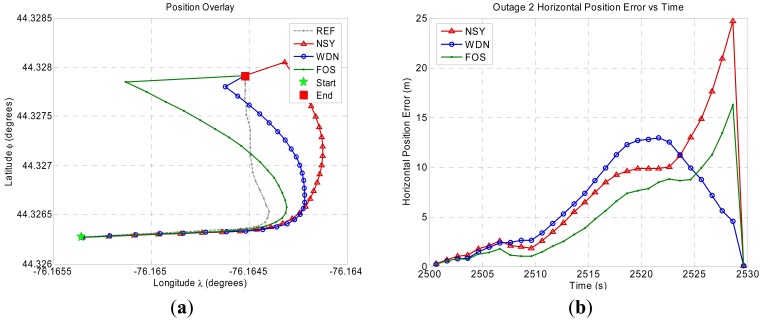
Land vehicle experiment position domain results for GPS outage 2. (**a**) Position domain plot. (**b**) Horizontal position error plot.

**Figure 10.**
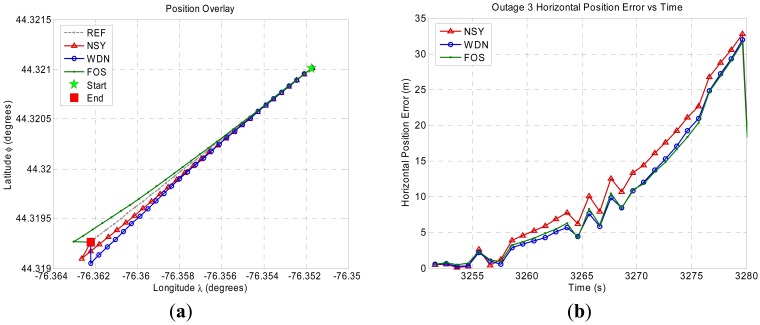
Land vehicle experiment position domain results for GPS outage 3. (**a**) Position domain plot. (**b**) Horizontal position error plot.

**Figure 11.**
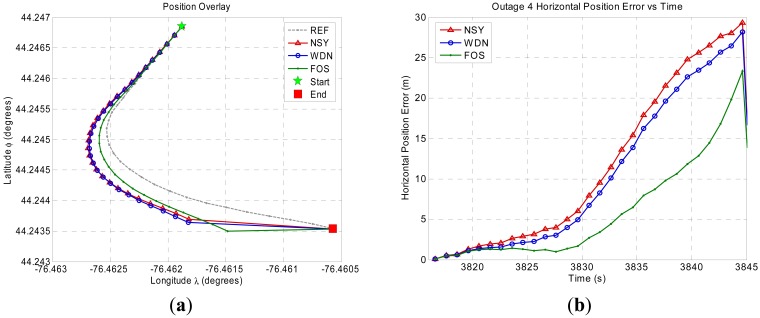
Land vehicle experiment position domain results for GPS outage 4. (**a**) Position domain plot. (**b**) Horizontal position error plot.

Figures 8–11 show the position domain results and the corresponding horizontal position errors over the duration of each of the four GPS outages. The maximum and mean horizontal position errors during the four GPS outages for the NSY, WDN and FOS trajectories are summarized in Table 5. The percentage improvement over NSY provides an indication of the accuracy enhancement performance in the position domain. Considering the average maximum horizontal position error values, FOS accuracy enhancement was slightly better than wavelet de-noising. However, the mean horizontal position error values indicate that FOS provided accuracy enhancement during the GPS outages that was relatively superior to wavelet de-noising. For example, it can be noticed in Figure 9 for GPS outage # 2 that the position errors in case of FOS was less than WDN for the whole GPS outages except for the last few seconds when the vehicle made a sharp turn. In this case, the maximum position error for FOS was slightly larger than WDN while the mean position error over the whole GPS outage showed better FOS performance over WDN [23]. The main reason for this is the ability of FOS to extract the frequency components of the motion dynamics within the low frequency range where these dynamics are mixed with the long-term inertial sensor errors. Wavelet, on the other hand, was only able to attenuate high frequency noises and some of short term errors.

The results verify that FOS is capable of processing real IMU data from a relatively long trajectory of one hour in duration. The FOS output is noticeably less noisy than the NSY signals for all sensors, and it is even less noisy than the WDN signals in the case of the X and Y gyroscopes (not shown in this paper). The residual frequency components above the estimated motion dynamics frequency range that are present in the WDN signals may be attributed to the thresholding techniques used in wavelet de-noising. Due to the setting of the parameters, FOS did not include these frequency components when it synthesized the estimate of the vehicle motion dynamics. These additional components do not likely constitute a significant portion of the true motion dynamics since they are outside the expected frequency range for land vehicle motion, but they could be attributed to road surface irregularities or vibrations from other machinery within the vehicle (*i.e.*, pumps, fans, *etc.*).

The NSY, WDN, and FOS position error values during the artificial GPS outages were in the order of those observed in other experimental work [23] with tactical grade IMUs and 30 s GPS outages. The maximum and mean horizontal position error values from FOS were generally comparable or superior to those from wavelet de-noising, with the exception of GPS outage 2. Overall, this experimental work demonstrates that FOS can contribute to accuracy enhancement of the vehicle position during GPS outages. In the case of GPS outage 2, the maximum horizontal position error for FOS is larger than WDN. However, the mean horizontal position error for FOS is smaller than WDN, which is still a significant achievement in accuracy enhancement.

### 5.2. MEMS IMU

**Figure 12.**
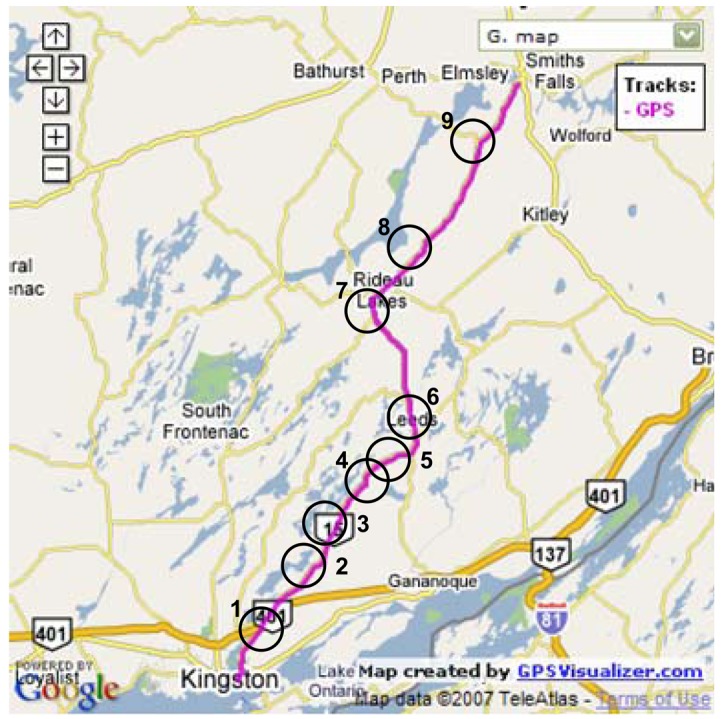
The second road test trajectory (for MEMS based IMU) with the location of some intentionally introduced GPS outages.

**Figure 13.**
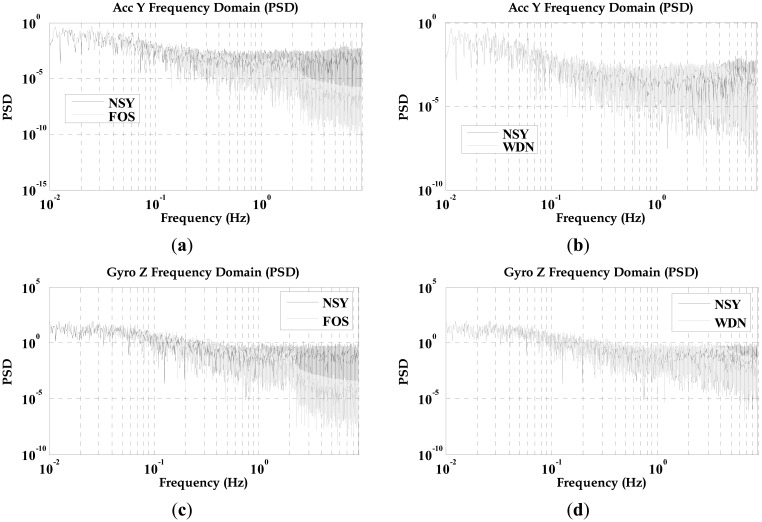
PSD plots for MEMS inertial sensors before and after applying both FOS and WDN.

**Figure 14.**
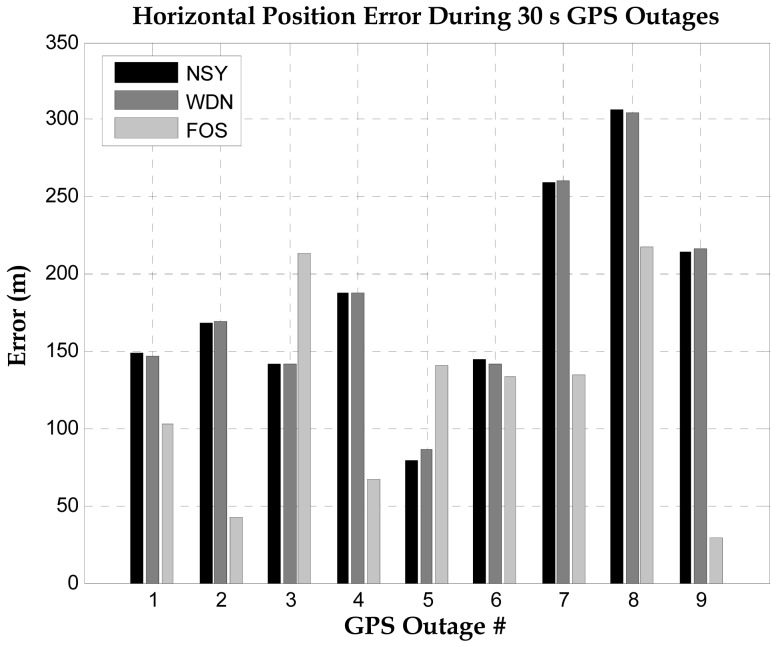
Positioning accuracy at the end of 9 GPS outages for MEMS IMU for the 2nd road test.

The inertial system used in the second road test is the Crossbow MEMS grade IMU (Crossbow Technologies) with its specifications shown on Table 4. The trajectory of the second road test is shown in Figure 12. This experiment was performed between the City of Kingston and Smith Falls (Ontario, Canada; see Figure 12) of and the inertial sensor measurements were acquired at 20 Hz. The raw sensor measurements were processed by both WDN and FOS and the PSD plots for the forward accelerometer and the vertical gyroscopes are shown in Figure 13. It can be noticed from these plots that FOS attenuated some of the noises in the frequency band containing the motion dynamics, while WDN was only able to attenuate frequencies outside this frequency band. The influence of WDN appears only after 4 Hz. We introduced 9 artificial GPS outages, each of 30 s, at different locations along the trajectory as can be seen in Figure 12. The performance of the overall system before and after pre-filtering was assessed in terms of the position error at the end of the GPS outage. The results are shown in Figure 14 for the 9 GPS outages and for the NSY, WDN and FOS signals and more details are given in Table 6.

Clearly the overall system performance benefited from the de-noising by FOS and position errors was reduced after the pre-filtering process. Moreover, except for GPS outages 3 and 5, FOS provided better accuracies than wavelet. In fact, WDN was providing almost the same accuracy of the raw noisy signal for all GPS outages. The average percentage improvement (considering all 9 GPS outages) for the vehicle horizontal position was 24% when using FOS and almost no improvement when using wavelets. In fact the superior accuracy obtained with FOS de-noising is due to the ability of FOS to fit the frequencies with the highest energy first, which are mostly the motion dynamic terms, and then reject terms (the long term errors or insignificant motion dynamics) with its adaptive noise threshold. Such errors exist in the very low frequency part of the signal and mixed with motion dynamics, thus cannot be removed by wavelet de-nosing since it is basically a lowpass filtering and do not include the noise rejection threshold inherent in FOS.

## 6. Conclusions

The experimental work discussed in this section for tactical grade IMUs validates FOS as an inertial sensor accuracy enhancement technique that is applicable to a real INS integrated with a GPS and applicable to relatively long trajectories. Processing raw inertial measurements with FOS can contribute to improved accuracy enhancement in the position domain during GPS outages, and the position improvements are frequently better than wavelet de-noising. For tactical grade IMUs, the mean horizontal position error was reduced by 23% after applying FOS while wavelet de-nosing has only shown 10% enhancement. For MEMS-based IMU, FOS has shown 74% improvement while wavelet has only shown 55%. The main reason of the superior accuracy provided by FOS is its capability of removing the long-term inertial sensor errors prior to INS mechanization and KF-based integration with GPS.

## Figures and Tables

**Table 1.** Wavelet de-noising parameters.

**Signal**	**Wavelet**	**LOD**	**Threshold Parameters**

x-gyro, y-gyro	Daubechies 1 (db1)	10	Soft, SURE, No Rescaling
z-gyro	Daubechies 3 (db3)	6	Soft, SURE, No Rescaling
x-acc, y-acc	Daubechies 3 (db3)	6	Soft, SURE, No Rescaling
z-acc	Daubechies 1 (db1)	10	Soft, SURE, No Rescaling

**Table 2.** FOS de-noising parameters.

**Signal**	**Candidate Functions (Hz)**	**Threshold**	**MAXFTA**	**Synthesis Range**

x-gyro, y-gyro	0–0.0366 @ 1/8 FFT Res	4% var [NSY]	6	0–0.0366
z-gyro	0–0.6 @ 1/8 FFT Res	4% var [NSY]	6	0–0.6
x-acc, y-acc	0–0.6 @ 1/8 FFT Res	4% var [NSY]	6	0–0.6
z-acc	0–0.0366 @ 1/8 FFT Res	4% var [NSY]	6	0–0.0366

**Table 3.** Summary of position domain accuracy enhancement.

	**NSY**	**WDN**	**FOS**

**Maximum Horizontal Position Error (m)**	204.0	192.6	27.8
**Improvement over NSY (%)**	-	5.6%	86.4%
**Mean Horizontal Position Error (m)**	118.8	110.4	17.5
**Improvement over NSY (%)**	-	7.1%	85.2%

**Table 4.** Specifications for TG-6000 IMU and Lassen SQ GPS module.

	**TG-6000 IMU**	**MEMS Crossbow IMU**	**Lassen SQ GPS Module**
**Manufacturer**	KVH Industries Inc., Middletown, RI	Crossbow Technologies	Trimble Navigation Ltd., Sunnyvale, CA
**Performance**	*Gyroscopes* Input Range: ±750°/s Bias drift: ±10°/h *Accelerometers* Input Range: ±7 g Bias drift: ±10 mg/h	*Gyroscopes* Input Range: ±100°/s Bias drift: ±2°/s *Accelerometers* Input Range: ±2 g Bias drift: ±30 mg/h	Operational limits: <18,000 m or velocity <515 m/s *Accuracy* Horizontal: <6 m (50%), <9 m (90%) Vertical (Altitude): <11 m (50%), <18 m (90%) Velocity: 0.06 m/s *Acquisition* Reacquisition: <2 s (90%) Cold Start: <90 s (50%), <170 s (90%)
**Interface (Digital)**	Data Rate: up to 150 Hz RS-422 serial 115,200 baud	Data Rate: up to 200 Hz RS-232 serial 9,600 baud	Update Rate: 1 Hz RS-232 serial 9,600 baud
**Power**	Input Voltage: 14 to 30 VDC, 12 Watts max.	Input Voltage: 9 to 30 VDC, 3 Watts max.	Input Voltage: 3.0 to 3.6 VDC, 0.5 Watts max.
**Miscellaneous**	75 Hz sampling rate during data acquisition	20 Hz sampling rate during data acquisition	Uses 3 V active micropatch antenna with magnetic mount

**Table 5.** Summary of position domain accuracy enhancement during GPS outages (Road Test 1).

	**Outage 1** **(1,371–1,400 s)**	**Outage 2** **(2,500–2,529 s)**	**Outage 3** **(3,251–3,280 s)**	**Outage 4** **(3,816–3,845 s)**	**Average** **(All Outages)**

**NSY Maximum Horizontal Position Error (m)**	38.8	24.7	32.8	29.3	31.4
**WDN Maximum Horizontal Position Error (m)**	37.4	12.9	31.9	28.2	27.6
**WDN Enhancement (%)**	4%	48%	3%	4%	14%
**FOS Maximum Horizontal Position Error (m)**	36.3	16.3	31.6	23.3	26.9
**FOS Enhancement (%)**	7%	34%	4%	20%	16%
**NSY Mean Horizontal Position Error (m)**	19.4	9.5	14.8	15.2	14.7
**WDN Mean Horizontal Position Error (m)**	18.7	7.5	13.4	14.0	13.4
**WDN Enhancement (%)**	3%	21%	9%	8%	10%
**FOS Mean Horizontal Position Error (m)**	18.1	6.6	13.3	8.6	11.6
**FOS Enhancement (%)**	6%	31%	10%	43 %	23%

**Table 6.** Summary of percentage position improvement during GPS outages (Road Test 2).

**Outage #**	1	2	3	4	5	6	7	8	9

**WDN (%)**	1.7	0.4	0.0	0.0	−8.4	2.3	0.1	0.8	0.7
**FOS (%)**	31.3	74.7	−50.6	64.2	−77.8	7.6	48.2	29.3	86.4
